# Health facility-based prevalence of typhoid fever, typhus and malaria among individuals suspected of acute febrile illnesses in Southwest Region, Ethiopia

**DOI:** 10.3389/fepid.2024.1391890

**Published:** 2024-07-18

**Authors:** Mengistu Abayneh, Mitiku Aberad, Yosef Habtemariam, Yared Alemu

**Affiliations:** ^1^ School of Medical Laboratory Sciences, Faculty of Health Science, Institute of Health, Jimma University, Jimma, Ethiopia; ^2^ College of Medical and Health Science, Department of Medical Laboratory Sciences, Mizan-Tepi University, Mizan Teferi, Ethiopia; ^3^ College of Medical and Health Science, Department of Medicine, Mizan-Tepi University, Mizan Teferi, Ethiopia

**Keywords:** seroprevalence, typhoid fever, typhus, malaria, Southwest Ethiopia

## Abstract

**Background:**

Acute febrile illnesses such as typhoid fever, typhus, and malaria are still major causes of hospital admission in many parts of Ethiopia. However, there are substantial gaps in the monitoring systems, which result in a lack of knowledge about the geographic distribution and role of common pathogens, particularly in rural areas. Thus, this study was aimed at assessing the seroprevalence of typhoid fever, typhus, and malaria among suspected acute febrile patients at the MTU Teaching Hospital and Mizan-Aman Health Center, Southwest region of Ethiopia.

**Method:**

A health facility-based cross-sectional study was carried out from July to October 2022. Blood samples were collected from a total of 384 individuals. Widal and Weilfelix direct card agglutination and tube agglutination test methods were used for the *Salmonella enterica* serotype *Typhi* (*S. typhi*) and *Rickettsia* infections. The diagnosis of malaria was made using thick and thin blood smears. Questionnaires given by interviewers were used to gather information on risk factors and other sociodemographic factors. The data was analyzed using STATA/SE 14.0.

**Result:**

A total of 371 patients were tested for *S*. Typhi and *Rickettsia* infections using direct card agglutination and tube agglutination methods. Using the screening test, 20.5% (76/371) patients were reactive either for O or H antigens or both, of which 55.3% (42/76) were reactive by the titration test at the cutoff value ≥ 1:80. About 17.5% (65/371) were reactive to OX19 antigen by card agglutination test, and of which 58.5% (38/65) were reactive by the titration test at the cutoff value ≥ 1:80. The overall seroprevalence of *S*. Typhi and *Rickettsia* infections using combined direct card and tube agglutination techniques was 11.3% (42/371) and 10.2% (38/371), respectively. Out of 384 suspected malaria patients, 43 (11.2%) were found positive either for *P*. *falciparum,* 27 (7.03%), or *P*. *vivax,* 16 (4.2%).

**Conclusion:**

In this study, typhoid fever, typhus, and malaria were found among symptomatic acute febrile patients. To increase disease awareness, it is necessary to provide sustainable health education about risk factor behaviors, disease transmission, and prevention strategies. In addition, improving laboratory diagnosis services and early treatment may also lower the likelihood of potentially fatal consequences.

## Background

Although there has been tremendous progress over the past decade, febrile illnesses such as malaria and acute bacterial infections such as pneumonia, typhoid fever, typhus, and relapsing fever are the major causes of seeking healthcare and may be responsible for varying degrees of morbidity and mortality, especially in sub-Saharan Africa (1). A study in East Africa reported that the pooled prevalence of febrile cases with unidentified etiology was 64%, with a prevalence of 69% in Ethiopia, 67% in Kenya, and 61% in Tanzania (2). These diseases are often known by non-specific clinical signs and symptoms, and the scarcity of the proper diagnostic tools and limited laboratory capacity are the major challenges for health professionals in the diagnosis and treatment of patients (1–3).

The incidence of febrile illness and the prevalence of various pathogens vary by factors such as location, season, and urban or rural setting. Such infections like malaria, typhoid fever, and typhus share similar predisposing factors such as poverty, poor sanitation, and public health services, and in the same way, individuals in areas endemic for these infections are being put at substantial risk of contracting concurrently (4–6). In addition, although early and accurate diagnosis of fevers is essential for effective case management, due to the limited availability of diagnostics in low- and middle-income countries, most cases of acute febrile illnesses with symptoms such as fever, headache, joint pain, and back pain are often misdiagnosed as malaria, or as typhoid fever, or typhus (3, 6–8), and management remains challenging, especially for non-malarial fever (3, 9). Consequently, treatable bacterial infections were missed, and antibiotics are overused or poorly targeted, that may be resulted for poor patient outcomes.

In Ethiopia, the data from the nationwide inventory of sanitation facilities reported that, more than half of the population still used unimproved sanitation facilities (10). However, few studies have been done to determine the prevalence and etiology of febrile illness among people who present with typical symptoms in some parts of the country (11–13), which emphasizes the significant knowledge gaps regarding the geographic distribution and role of common pathogens of most febrile illnesses, including typhoid fever, typhus, and malaria. The recommendations from the research output are very important for ensuring that every patient receives the right care at the right time and for proposing appropriate services or interventions. Therefore, in the selected population, we evaluated the prevalence of typhoid fever, typhus, and malaria in those who were complaining of symptoms like fever, headache, joint pain, and back pain in the selected health facilities in the Southwest region of Ethiopia.

## Methods and materials

### Study area and period

This study was conducted at Mizan-Tepi University (MTU) teaching hospital and Mizan-Aman health center from July to October 2022. The teaching hospital and the health center are found in Mizan-Aman town, at 591 Km in the Southwest direction, from Addis Ababa, the capital city of Ethiopia. These health facilities were providing services to approximately 5 million people coming from four catchment zones, such as BENCH SHEKO, KEFA, SHEKA and MAJANG.

### Study design and subjects

A cross-sectional study was conducted to determine the prevalence of typhoid fever, typhus, and malaria among individuals who were complaining of a range of symptoms. All adults and children ≥2 years old and presenting with acute febrile illness at MTU Teaching Hospital and Mizan-Aman Health Center outpatients and emergency departments and willing to provide blood and gave informed consent and/or assents were included in the study. The acute fever may be measured when the patient presents, or it can be a recent history of fever (a body temperature ≥ 37.5°C with in the last 7 days), and the fever can be with or without other many possible symptoms, but generally not a localized infection. We didn't include inpatients, chronic fever, and new-borns or very young children (e. g., <2 years).

### Sample size and sampling techniques

The required sample size is calculated using Cochran's general formula for a single population proportion by assuming a confidence interval of 95%, a margin error of 5%, and a prevalence of 0.5. Hence, the minimum number of study participants that were enrolled in the study was 384, and the required data were collected from consecutive patients during the study period.

### Data collection process

#### Socio-demographic and other risk factor data

An interviewer administered questionnaires was used to collect the data. The questioner was developed after reviewing similar research conducted in Ethiopia and abroad. Socio-demographic data such as age, sex, educational status, occupational status, and clinical-related data such as body temperature, headache, joint pain, back pain, illness duration, and contact history with the same symptoms, and environmental related data such as source of water, availability of toilet facilities, hand washing habit, and raw meat and raw vegetable consumption habit, were collected. Knowledge of study participants of age ≥ 15 years on the causes, symptoms, ways of transmission, and prevention methods Typhoid fever, typhus, and malaria diseases were assessed. Three data collectors were assigned to collect the data after a briefing on the objective and purpose of the study. Once the appropriate history and information were collected, laboratory samples were collected. The questionnaire was checked daily for completeness, accuracy, clarity, and consistency, and necessary corrections were made.

### Collection and processing of blood samples

The blood samples were collected from all febrile individuals after informed consent was obtained. Three milliliters (3 ml) of venous blood were collected from each patient into an EDTA® vacutainer test tube by the skilled laboratory technologist. But 13 patients were provided finger prick blood samples due to the obscurity of veins. First, thin, and thick blood smears were prepared with a small drop of blood for the detection of malaria. Then, following the manufacturer's instructions and as previously described (14, 15), the serum was separated, and on the same days, screening tests for *Salmonella* and *Rickettsia* infections were conducted using the Widal and Weil-felix direct card agglutination test techniques. All serum samples that were found to be reactive by the direct card agglutination tests were transported to the MTU microbiology laboratory and further tested by the tube agglutination test method. In detail, the serum samples were serially diluted using a fresh 0.95% saline preparation from 1:20 to 1:5,120 for anti-O, anti-H, and anti-OX19 separately in 10 test tubes. After adding one drop of O, H, and OX19 antigens to each test tube, it was incubated for 2–4 h at 50°C. An antibody titter of 1:80 for anti-O, for anti-H and anti-OX19 antibodies was taken as a cut-off value to indicate recent typhoid and *Rickettsia* infections. For quality assurance, positive and negative control of Widal and Weilfelix direct card agglutination tests were performed each day before taking to do patient's sample according to manufacturer instructions.

### Data analysis and interpretation

The data was entered and analyzed using STATA/SE 14.0. Descriptive analysis, such as frequencies and percentages of variables, was used to summarize the data. Pearson chi-square test was performed to evaluate the statistically significant difference in the prevalence of typhoid fever, typhus, and malaria between the demographics of the study participants and according to their reported clinical and environmental features. Bivariate and multivariate logistic regression analyses were performed to assess the associations of socio-demographic, clinical characteristics, and hygienic or environmental conditions of the study participants with increased odds of having a higher prevalence of typhoid fever, typhus, and malaria. A *p*-value below 5% was considered as indicator of statistical significance.

## Results

### Socio-demographic characteristics

A total of 384 individuals who fulfilled acute febrile illness (AFI) criteria participated in the study. Of these, female patients account for 57.6% (*n* = 221), and male account for 42.4% (*n* = 163). The age range was 2–75 years, with a mean age of 27.64 years. All individuals were volunteers to consent to provide a venous blood sample, but 13 patients were provided a finger- prick blood sample due to the invisibility of their veins. The majority (65.2%) were from rural area and had only attained primary school. and were farmers/housewives in their occupations (Table 1).

**Table 1:** Socio-demographic characteristics of the study participants.

Variable	Category	Number tested	Percent
Sex	Male	163	42.4%
	Female	221	57.6%
Age in years	2–14	112	29.2%
	15–24	70	18.2%
	25–34	65	16.9%
	35–44	65	16.9%
	45–54	52	13.5%
	≥55	20	5.2%
Residence	Urban	151	39.3%
	Rural	233	60.7%
Occupation	Farmer/housewife	215	56.0%
	Civil servant	57	14.8%
	Merchant	39	10.2%
	Other	73	19.0%
Educational status	No education	129	33.6%
	Primary education	193	50.3%
	Secondary and above	62	16.1%

In this study, all patients with an age group of ≥15 years were assessed for their knowledge about the causes of infections, symptoms of the disease, ways of transmission, and prevention methods of typhoid fever, typhus, and malaria. The patients were categorized as having good knowledge if he/she got ≥50% of the answer from each category of the questions. Accordingly, only 63 (24.1%) and 42 (16.1%) of individuals have good knowledge about typhoid fever and typhus infections, respectively. Only 78 (28.7%) had good knowledge about malaria infection.

### The prevalence of typhoid, typhus, and malaria

**Figure 1:**
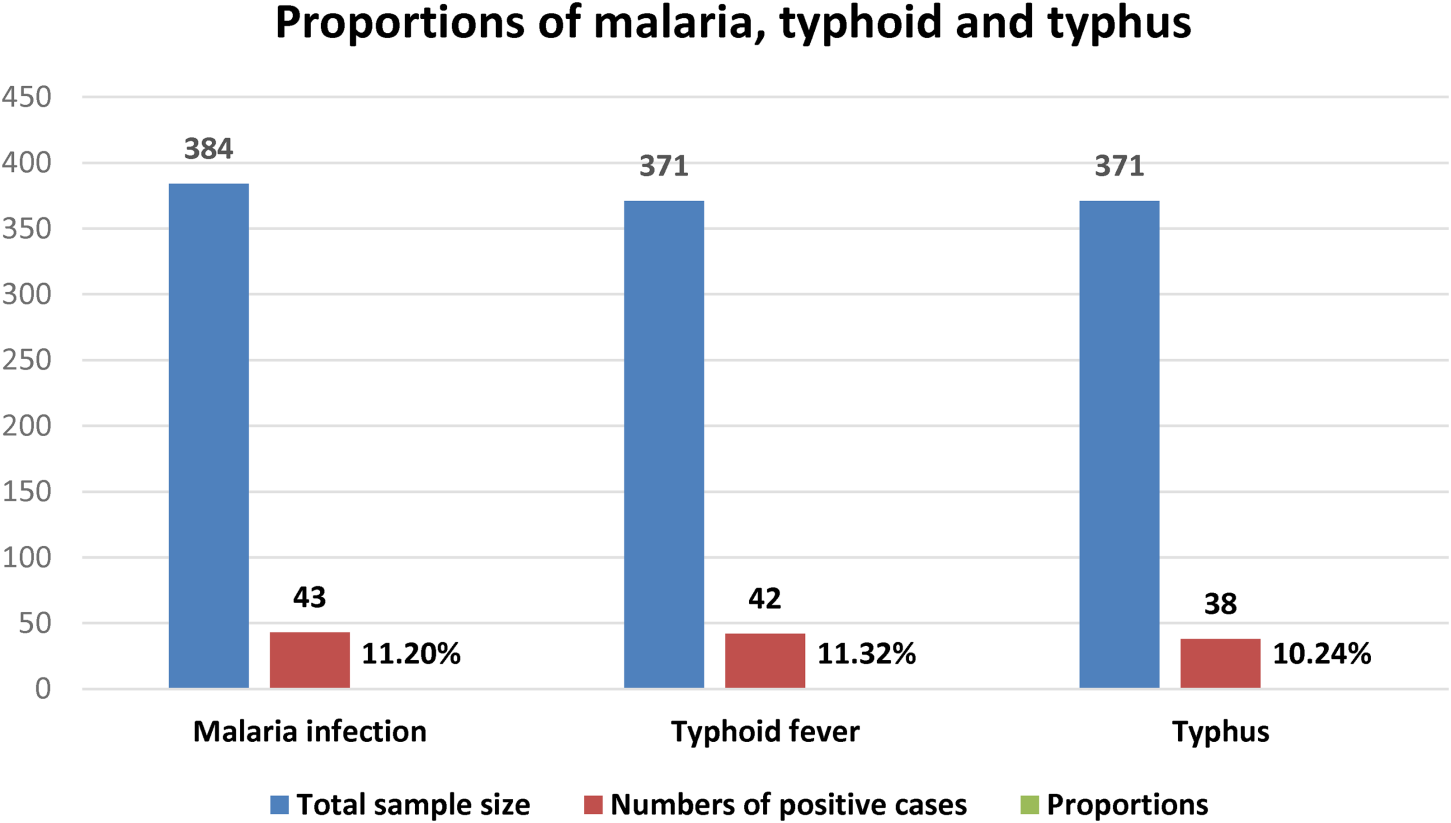
Proportions of typhoid, typhus, and malaria infections.

Only 43 (11.2%) of the 384 febrile patients tested microscopically positive for both *P. falciparum* and *P. vivax*. Among a total of 371 febrile patients, 20.5% (76/371) and 17.5% (65/371) were reactive for S. Typhi and *Rickettsia* infection by using the screening Widal and Weilfelix direct card agglutination test. Of these, 42 (11.3%) and 38 (10.2%) were positive for S. Typhi and *Rickettsia* infections as tested by titration techniques, respectively (Figure 1).

### Prevalence of *S*. typhi infection

A total of 371 patients were tested for *S*. typhi infection using direct card agglutination and tube agglutination methods. During the screening test, 20.5% (76/371) of the patients were reactive against somatic (O) or flagella (H) antigens or both. In detail, 29/76 (38.2%) were reactive only against O antigen, 24 (31.6%) against flagella (H) antigen and 23 (30.3%) against both H, and O antigens. The total sera reactive either against O and/or H antigen by the tube agglutination test was 55.3% (42/76) at the cut off value of 1:80. Of these, 13 (17.1%), 7 (9.20%), and 3 (3.9%) of the reactive sera to O antigen were reactive at a titration of 1:80, 1:160, and 1:320, respectively. Among the reactive sera to H antigen, 11 (14.5%), 5 (6.6%), and 3 (3.9%) were reactive at the titration of 1:80, 1:160 and 1:320, respectively. Hence, the total seroprevalence of *S*. Typhi infection using direct card agglutination and tube agglutination technique was 11.3% (42/371) (Figure 1).

The prevalence of *S*. typhi infection was significantly higher among females (14.0%; AOR = 2.25; 95% CI: 1.01–5.05, *P* *= *0.049) than among males (7.6%), as detected by the titration tests. Patients with no formal education (AOR = 3.82, 95%CI: 1.06–13.78, *P* = 0.041) and those with clinical symptoms of joint and/back pain (AOR = 3.89, 95%CI: 1.05–14.36, *P* = 0.042) were found to be associated with a higher rate of *S*. Typhi infection (Table 2).

**Table 2:** Socio-demographic, clinical characteristics and hygienic conditions of the study participants and prevalence of typhoid fever infection.

Variable	Category	Number tested	Positives by titration (%)	AOR (95%CI)	*P*-value
Sex	Male	157	12 (7.6)	1	
	Female	214	30 (14.0)	2.25 (1.01, 5.05)	0.049*
Age	2–14	110	15 (13.6)	0.73 (0.15, 3.67)	0.707
	15–24	66	6 (9.1)	0.54 (0.09, 3.16)	0.497
	25–34	63	7 (11.1)	0.75 (0.13, 4.21)	0.741
	35–44	62	5 (8.1)	0.46 (0.08, 2.74)	0.393
	45–54	50	6 (12.0)	0.86 (0.14, 5.17)	0.866
	≥55	20	3 (15.0)	1	
Residence	Urban	145	16 (11.0)	1	
	Rural	226	26 (11.5)	1.04 (0.46, 2.34)	0.926
Occupation	Farmer/housewife	207	24 (11.6)	1.34 (0.41, 4.43)	0.628
	Civil servant	54	5 (9.3)	1	
	Merchant	39	3 (7.7)	0.85 (0.15, 4.95)	0.857
	Other	71	10 (14.1)	1.27 (0.32, 5.11)	0.735
Educational status	No education	124	22 (17.7)	3.82 (1.06, 13.78)	0.041*
	Primary education	187	16 (8.6)	1.33 (0.38, 4.70)	0.655
	Secondary and above	60	4 (6.7)	1	
Body temperature	37.5–38°C	322	37 (11.5)	1	
	>38°C	49	5 (10.2)	0.79 (0.25, 2.44)	0.678
Headache	No	72	7 (9.7)	1	
	Yes	299	35 (11.7)	0.94 (0.32, 2.82)	0.919
Joint and/back pain	No	87	3 (3.4)	1	
	Yes	284	39 (13.7)	3.89 (1.05, 14.36)	0.042*

*Variables having statistical significance association.

**Table:** 

Variable	Category	Number tested	Positives by titration (%)	AOR (95%CI)	*P*-value
Contact history with the same symptoms	No	307	36 (11.7)	1	
	Yes	64	6 (9.4)	0.82 (0.29, 2.29)	0.700
Illness duration	≤4 days	331	38 (11.5)	1	
	5–7 days	40	4 (10.0)	0.52 (0.14, 1.85)	0.311
Knowledge of study participants of age >15 years	Good	63	3 (6.4)	1	
	Poor	198	24 (12.1)	2.89 (0.79, 10.51)	0.108
Source of water	Public/private tap water	91	6 (6.6)	1	
	Piped water	88	6 (6.8)	1.03 (0.24, 4.46)	0.971
	Protected well/spring	72	5 (6.9)	0.96 (0.23, 4.01)	0.953
	Unprotected well/spring	120	25 (20.8)	3.30 (1.01, 10.83)	0.049*
Availability of functional toilet	Yes	127	14 (11.0)	1	
	No	244	28 (11.5)	0.57 (0.22, 1.43)	0.229
Hand washing after toilet	Always	110	11 (10.0)	1	
	Sometimes	261	31 (11.9)	0.79 (0.33, 1.89)	0.592
Raw meat consumption habit	No	158	10 (6.3)	1	
	Yes	213	32 (15.0)	3.22 (1.09,9.48)	0.034*
Raw vegetable consumption habit	No	63	7 (11.1)	1	
	Yes	308	35 (11.4)	0.47 (0.15, 1.51)	0.115
Treatment of water before drinking	Yes	33	2 (6.1)	1	
	No	338	40 (11.8)	1.29 (0.21, 7.95)	0.781
Washing vegetable and fruit before eating	Yes	122	7 (5.7)	1	
	No	249	35 (14.1)	1.60 (0.56, 4.60)	0.378

Regarding the hygienic conditions of the study participants and *S. typhi* infection, patients who used unprotected well/spring water as a water source were 3.30 times (AOR = 3.30, 95%CI: 1.01–10.83, *P* = 0.049) more likely to get infection than those who used public/private tap water. Those patients with raw meat consumption habits are 3.22 times (AOR = 3.22, 95% CI: 1.09–9.48, *P* = 0.034) more likely to get *S*. typhi infection than non- consumers (Table 2).

### Prevalence of *Rickettsia* infection

In this study, a total of 371 patients’ serum was screened for *Rickettsia* infection by direct card agglutination, and 17.5% (65/371) were reactive against the OX19 antigen. Out of these reactive sera, 22 (33.8%), 10 (15.4%), and 6 (9.2%) were reactive at a titration of 1:80, 1:160, and 1:320, respectively. Hence, the total seroprevalence of *Rickettsia* infection using direct card agglutination and tube agglutination technique, was 10.2% (38/371).

The rate of infections was more significant among females (AOR = 2.31; 95% CI: 1.04–5.10, *P *= 0.039) and those who had contact history of the same symptoms (AOR = 2.71; 95% CI: 1.17–6.26, *P *= 0.020) as compared to males and those who do not have contact history as done by titration test (Table 3).

**Table 3:** Socio-demographic and clinical characteristics and prevalence of typhus infection.

Variable	Category	Number tested	Positives by titration (%)	AOR (95%CI)	*P*-value
Sex	Male	157	10 (6.4)	1	
	Female	214	28 (13.1)	2.31 (1.04, 5.10)	0.039*
Age	2–14	110	12 (10.9)	1.89 (0.34, 10.66)	0.470
	15–24	66	6 (9.1)	1.20 (0.19, 7.49)	0.846
	25–34	63	8 (12.7)	1.69 (0.28, 10.14)	0.568
	35–44	62	4 (6.5)	0.70 (0.10, 4.74)	0.717
	45–54	50	6 (12.0)	1.74 (0.26, 11.61)	0.565
	≥55	20	2 (10.0)	1	
Residence	Urban	145	12 (8.3)	1	
	Rural	226	26 (11.5)	1.52 (0.66, 3.52)	0.327
Occupation	Farmer/housewife	207	21 (10.1)	1.18 (0.36, 3.84)	0.786
	Civil servant	54	4 (7.4)	1	
	Merchant	39	3 (7.7)	1.28 (0.25, 6.56)	0.771
	Other	71	10 (14.1)	2.14 (0.57, 8.03)	0.258
Educational status	No education	124	16 (12.9)	2.50 (0.70, 8.91)	0.15 7
	Primary education	187	18 (9.6)	1.96 (0.57, 6.76)	0.285
	Secondary and above	60	4 (6.7)	1	
Body temperature	37.5–38°C	322	32 (9.9)	1	
	>38°C	49	6 (12.2)	1.05 (0.38, 2.94)	0.920
Headache	Yes	299	31 (10.4)	0.91 (0.38, 2.38)	0.853
	No	72	7 (9.7)	1	
Joint/back pain	Yes	284	30 (10.6)	1.36 (0.54, 3.44)	0.515
	No	87	8 (9.2)	1	
Contact history with the same symptoms	Yes	64	13 (20.3)	2.71 (1.17, 6.26)	0.020*
	No	307	25 (8.1)	1	
Illness duration	≤4 days	331	35 (10.6)	1	
	5–7 days	40	3 (7.5)	0.47 (0.12, 1.81)	0.273
Knowledge of study participants of age >15 years	Good	42	4 (9.5)	1	
	Poor	219	22 (10.1)	0.94 (0.32, 2.76)	0.909

*Variables having statistical significance association.

### Prevalence of malaria infection

In this study, a total of 384 suspected malaria patients were microscopically tested for plasmodium infections, and 43 (11.2%) were found positive for *P*. *falciparum,* 27 (7.03%), and *P*. *vivax* [16 (4.2%)]. The rate of infection was significantly higher among females (14.0%) and among patients with body temperature > 38°C (20.4%). On binary regression analysis, being female (AOR = 2.15, 95% CI: 1.03–4.49, *P* = 0.042) and having fever > 38°C (AOR = 2.84, 95% CI: 1.17–6.87, *P* = 0.021) were independently associated with increased odds of having *Plasmodium* infection (Table 4).

**Table 4:** Socio-demographic, clinical and environmental characteristics, and malaria infection.

Variable	Category	Number tested	Number of positive (%)	AOR (95%CI)	*P*-value
Sex	Male	163	12 (7.4)	1	
	Female	221	31 (14.0)	2.15 (1.03, 4.49)	0.042*
Age	2–14	112	13 (11.6)	0.59 (0.14, 2.54)	0.486
	15–24	70	7 (10.0)	0.45 (0.09, 2.14)	0.317
	25–34	65	9 (13.8)	0.73 (0.16, 3.29)	0.681
	35–44	65	6 (9.2)	0.45 (0.09, 2.12)	0.310
	45–54	52	5 (9.6)	053 (0.10, 2.76)	0.450
	≥55	20	3 (15.0)	1	
Residence	Urban	151	20 (13.2)	1	
	Rural	233	23 (9.9)	0.64 (0.31, 1.33)	0.232
Occupation	Farmer/housewife	215	24 (11.2)	0.73 (0.28, 1.83)	0.489
	Civil servant	57	8 (14.0)	1	
	Merchant	39	3 (7.7)	0.42 (0.09, 1.90)	0.258
	Other	73	8 (11.0)	0.49 (0.15, 1.56)	0.227

*Variables having statistical significance association.

**Table:** 

Variable	Category	Number tested	Number of positive (%)	AOR (95%CI)	*P*-value
Educational status	No education	129	17 (13.2)	2.41 (0.71, 8.22)	0.160
	Primary education	193	22 (11.4)	1.89 (0.59, 6.12)	0.285
	Secondary and above	62	4 (6.5)	1	
Body temperature	37.5–38°C	335	33 (9.9)	1	
	>38°C	49	10 (20.4)	2.84 (1.17, 6.87)	0.021*
Headache	Yes	311	33 (10.6)	0.89 (0.37, 2.13)	0.788
	No	73	10 (13.7)	1	
Joint/back pain	Yes	293	34 (11.6)	1.74 (0.73, 4.16)	0.212
	No	91	9 (9.9)	1	
Contact history with the same symptoms at home	Yes	66	7 (10.6)	1.06 (0.41, 2.69)	0.907
	No	318	36 (11.3)	1	
Illness duration =	≤4 days	341	40 (11.7)	1	
	5–7 days	43	3 (7.0)	0.60 (0.16, 2.18)	0.436
Frequent use of bed net	Yes	123	11 (8.9)	1	
	No	261	32 (12.3)	1.42 (0.69, 2.93)	0.338
Was the house sprayed with insecticide within 12 months	Yes	138	13 (9.4)	1	
	No	246	30 (12.2)	1.19 (0.67, 2.65)	0.409
Presence of stagnant water near the living house	No	125	13 (10.4)	1	
	Yes	259	30 (11.6)	1.13 (0.57, 2.25)	0.731
Knowledge of study participants of age >15 years	Good	78	9 (11.5)	1	
	Poor	194	21 (10.8)	0.64 (0.28, 1.49)	0.306

## Discussion

A health facility-based study was conducted to assess the prevalence of typhoid fever, typhus, and malaria among individuals who reported the sign/symptoms of fever, headache, joint pain, and back pain in the Southwest region of Ethiopia. In our study, of the total individuals suspected of acute febrile illness, 11.3% and 10.2% were confirmed to be sero-positive for *S*. typhi, and *Rickettsia* infection by confirmatory tests. The sero-prevalence of S. typhi infection in this study was slightly higher than the previous reports in Ethiopia (15, 16), and India (17), but lower than other findings in Ethiopia (18, 19) and Sudan (20). The sero-prevalence of *Rickettsia* infection was in line with other reported findings in different developing countries (21–25), but it is lower than a finding in Ethiopia (15, 18), Malaysia (26), and India (27). This divergence in the results may rest on the differences in investigation methods, the study populations, and the awareness of the community about the transmission and prevention of *S*. typhi, and *Rickettsia* infections.

The distribution of typhoid risk factors is uneven within the sub-national boundary level and is geographically heterogeneous (4). In this study, the seroprevalence for *S*. typhi infection was significantly higher among females. In addition, level of education, use of unprotected well/spring water as their water sources and consumption of raw meat were significantly associated with a high rate of *S*. typhi infection. Similar results were reported in other previous studies in Ethiopia (4, 15, 28). Geographical location, inadequate food and personal hygiene, lack of potable water, and awareness of the community on the transmission and prevention of typhoid fever have been frequently cited as the major risk factors contributing to the high burden of *S*. typhi infection (29–31). As a result, the high seroprevalence of S. typhi infection in females may be related to their level of education and daily activities, such as the frequency of exposure to contaminated water while fetching or washing clothes. To lessen the burden of these diseases, it is crucial to provide access to safe water, create a solid healthcare system for the diagnosis and treatment of typhoid fever, and raise community awareness.

The sero-prevalence of *Rickettsia* infection was also significantly high among females and in patients who had contact history with family members who had the same sign/symptoms. Similar results were reported in other previous studies in other developing countries (22, 27, 32). Diagnostic services for non-malaria febrile cases are complex and not widely available, and with a few exceptions, preventive measures for specific infections are poorly developed or difficult to implement (33). Thus, implementation of an effective health care system for the diagnosis and treatment of *Rickettsial* infections and increasing community awareness on modes of acquisition and prevention strategies are very important to reduce the burden of these diseases.

The other finding of this study was a malaria prevalence of 11.2%, which is lower than the previous reported findings in Ethiopia (19, 34–38) and Nigeria (39). *P*. *falciparum* in this study was the most prevalent species that causes severe forms of malaria, as reported in previous studies (15, 34–36). Moreover, the prevalence of *Plasmodium* infection was significant among females and among patients with clinical signs of fever ≥ 37.5°C, as reported in previously conducted studies (2, 15, 34). Although Ethiopia has implemented a comprehensive program to control and prevent malaria, the prevalence of the disease has been rising in various regions of the nation since a few years ago. Hence, to achieve the plan for malaria elimination, it is crucial to improve case detection of malaria in this region and raise community understanding of the transmission and prevention of malaria, for instance through community health extension workers.

Serological agglutination assays are still used in developing countries because of their speed and technical ease of application, as well as their low cost (40). In this study, we also used Widal and Weil-felix tests to determine the seroprevalence of S. typhi and *Rickettsia* infections in those who had sign/symptoms of a febrile illness. Hence, one of the limitations of the current study may be that these serological-based tests do not provide conclusive evidence for the diagnosis of current infection because of flaws like false positivity due to prior exposure or false negativity for those living in an endemic setting (28, 40). Additionally, the selection of research participants may have been biased because it was based on clinical signs and symptoms that were reported by the participants rather than those that were necessarily brought on by an infectious agent that causes acute illness. Hence, the results of this study cannot be applied to other populations since, in an endemic area, people may have these diseases without exhibiting symptoms.

## Conclusion

Typhoid fever, typhus, and malaria were important infections observed among symptomatic acute febrile individuals in the study area. The sero-prevalence of each infection varies in relation to the socio-demographic, clinical characteristics, and hygienic or environmental conditions of the study participants. Therefore, efforts are needed to improve disease awareness by providing enhanced and sustainable health education about typhoid fever, typhus, and malaria infection, its transmission, prevention strategies, and identifying risky behaviours with focusing on households, housewives, and students. In addition, early diagnosis and treatment for these non-specific febrile illnesses is essential to reduce the incidence of potentially fatal complications.

## Data Availability

The original contributions presented in the study are included in the article/Supplementary Material, further inquiries can be directed to the corresponding author.
